# Epigenetic and transcriptomic alterations in offspring born to women with type 1 diabetes (the EPICOM study)

**DOI:** 10.1186/s12916-022-02514-x

**Published:** 2022-09-23

**Authors:** Sine Knorr, Anne Skakkebæk, Jesper Just, Emma B. Johannsen, Christian Trolle, Søren Vang, Zuzana Lohse, Birgitte Bytoft, Peter Damm, Kurt Højlund, Dorte M. Jensen, Claus H. Gravholt

**Affiliations:** 1 Steno Diabetes Center Aarhus, Aarhus University Hospital, Hedeager 3, 2. fl, 8200 Aarhus, DK Denmark; 2 Department of Molecular Medicine, Aarhus University Hospital, Aarhus, Denmark; 3 Department of Endocrinology and Internal Medicine, Aarhus University Hospital, Aarhus, Denmark; 4 Department of Clinical Genetics, Aarhus University Hospital, Aarhus, Denmark; 5 Steno Diabetes Center Odense, Odense University Hospital, Odense, Denmark; 6 Center for Pregnant Women with Diabetes, Department of Obstetrics, Rigshospitalet, Department of Clinical Medicine, University of Copenhagen, Copenhagen, Denmark

**Keywords:** Type 1 diabetes, Pregnancy, Epigenetics, Methylation, Transcriptome

## Abstract

**Background:**

Offspring born to women with pregestational type 1 diabetes (T1DM) are exposed to an intrauterine hyperglycemic milieu and has an increased risk of metabolic disease later in life. In this present study, we hypothesize that in utero exposure to T1DM alters offspring DNA methylation and gene expression, thereby altering their risk of future disease.

**Methods:**

Follow-up study using data from the Epigenetic, Genetic and Environmental Effects on Growth, Metabolism and Cognitive Functions in Offspring of Women with Type 1 Diabetes (EPICOM) collected between 2012 and 2013.

**Setting:**

Exploratory sub-study using data from the nationwide EPICOM study.

**Participants:**

Adolescent offspring born to women with T1DM (*n*=20) and controls (*n*=20) matched on age, sex, and postal code.

**Main outcome measures:**

This study investigates DNA methylation using the 450K-Illumina Infinium assay and RNA expression (RNA sequencing) of leucocytes from peripheral blood samples.

**Results:**

We identified 9 hypomethylated and 5 hypermethylated positions (*p* < 0.005, |Δ*M*-value| > 1) and 38 up- and 1 downregulated genes (*p* < 0.005, log2FC ≥ 0.3) in adolescent offspring born to women with T1DM compared to controls. None of these findings remained significant after correction for multiple testing. However, we identified differences in gene co-expression networks, which could be of biological significance, using weighted gene correlation network analysis. Interestingly, one of these modules was significantly associated with offspring born to women with T1DM.

Functional enrichment analysis, using the identified changes in methylation and gene expression as input, revealed enrichment in disease ontologies related to diabetes, carbohydrate and glucose metabolism, pathways including MAPK1/MAPK3 and MAPK family signaling, and genes related to T1DM, obesity, atherosclerosis, and vascular pathologies. Lastly, by integrating the DNA methylation and RNA expression data, we identified six genes where relevant methylation changes corresponded with RNA expression (*CIITA*, *TPM1*, *PXN*, *ST8SIA1*, *LIPA*, *DAXX*).

**Conclusions:**

These findings suggest the possibility for intrauterine exposure to maternal T1DM to impact later in life methylation and gene expression in the offspring, a profile that may be linked to the increased risk of vascular and metabolic disease later in life.

**Supplementary Information:**

The online version contains supplementary material available at 10.1186/s12916-022-02514-x.

## Background

The “Developmental Origins of Health And Disease” hypothesis describes how an adverse early life environment may lead to increased risk of adult disease [1]. Offspring born to women with pregestational type 1 diabetes (T1DM) are exposed to a hyperglycemic intrauterine milieu and recent studies have shown an increased risk of developing type 2 diabetes (T2DM), cardiovascular disease, and obesity [2–4]. The pathophysiological mechanisms behind this increased risk in the offspring are unclear, and whether a link to intrauterine T1DM exposure exists remains to be elucidated. Epigenetic modifications have been proposed as a possible mechanism by which maternal diabetes induces long-term effects [5]. However, our knowledge on epigenetic programming of offspring born to women with T1DM is still limited.

Methylation of cytosine is the best-studied epigenetic mark, with the addition of a methyl group to the C5 position of the cytosine-ring, and generally occurs on cytosines followed by a guanine (CpG). Genomic regions with an overrepresentation of CpG sites are termed CpG islands and are found at 60–70% of human gene promoters [6]. Hypermethylation of CpG islands is involved in transcriptional repression, thus, changes in DNA methylation adds level of regulation to the DNA molecule without changing the DNA sequence and via this mechanism DNA transcription can be altered [7]. Even though CpG sites are more sparse in gene bodies than in promotors, studies have described CpG hypermethylation in gene bodies to positively correlate with RNA expression [8]. Changes in DNA methylation is well known to be involved in cell differentiation and embryogenesis and is considered to be part of developmental biology [9]. Besides CpG methylation, epigenetic alterations also include histone modifications and non-coding RNA [5].

Recent studies in cord blood and peripheral blood have linked especially maternal overweight but also, to a lesser degree, maternal gestational diabetes (GDM) to offspring epigenetic alterations [10–13]. Few studies have evaluated the impact of maternal T1DM on offspring epigenetic changes, and included studies exploring an adult cohort including both offspring born to women with gestational diabetes and T1DM, using muscle, adipose tissue, or preadipocytes, described increased expression of microRNA-15a and microRNA-15b in skeletal muscle tissue and decreased leptin promotor methylation in subcutaneous adipocytes in offspring of T1DM women [12, 14–17]. Only one study by Gaultier et al. explored the more genome-wide DNA methylation profile using Illumina Human Methylation 27 Beadchip in describing an association between intrauterine exposure to maternal T1DM and kidney function in the adult offspring [18].

In this study, we hypothesized that in utero exposure to maternal T1DM alters global DNA methylation and RNA expression and thereby alters the offspring’s risk of later disease development.

## Methods

### The EPICOM cohort

This study is a part of the EPICOM (Environmental Versus Genetic and Epigenetic Influences on Growth, Metabolism and Cognitive Functions in Offspring of Women with Type 1 Diabetes) study (ClinicalTrials.gov registration no. NCT01559181). From 1992 to 1999, pregnancies in women with pregestational T1DM were prospectively reported to a registry managed by the Danish Diabetes Association. Information regarding diabetes status and pregnancy outcome was reported to the registry by local obstetricians at eight hospitals in Denmark, who were responsible for antenatal care and delivery for pregnant women with T1DM. Coverage of cases from the reporting centers spanned from 75 to 93%, as described by Jensen et al. [19]. The Danish Diabetes Association Register consists of 1326 records of children born to women with T1DM. Only children born after 24 completed gestational weeks were registered. As part of the EPICOM study, we invited one child per mother (the oldest) to participate in a clinical study concerning metabolic risk. Controls were recruited from the background population and were matched by sex, age, and postal number. Details from this study have previously been described by Vlachova et al. [20].

### Sample inclusion

Among the oldest participants of the EPICOM cohort, we selected 20 offspring of women with pregestational T1DM (index children) and their 20 matched controls (controls) as our exploratory cohort of DNA methylation and RNA expression (Table 1). Besides fulfilling the matching criteria for inclusion in the EPICOM cohort, we made sure that our exploratory cohort did not differ in body mass index (BMI), glucose tolerance, HbA_1c_, cholesterol, or triglyceride levels and that the index child and their control were age matched. The index/control pairs were chosen representing the pairs with the smallest age difference, living in the same postal code, gender matched, and not suffering from any chronical illnesses. DNA methylation and RNA expression profiling were performed on leucocytes isolated from peripheral blood samples.

**Table 1:** Baseline characteristics of diabetes-exposed children and controls

	Exposed to T1DM	Control children	*P*-value
Number of children	20	20	
Male sex	10 (50%)	10 (50%)	
Age (years)	17.8 (0.8)	18.1 (0.8)	0.26
Body mass index (kg/m^2^)	23.2 (2.6)	23.4 (2.2)	0.81
Oral glucose tolerance test (mmol/L)			
Time = 0 min	5.4 (0.4)	5.3 (0.3)	0.60
Time = 120 min	6.0 (0.9)	6.4 (0.8)	0.20
S-Insulin (fasting) (pmol/L)	55.8 (17.3)	57.9 (19.8)	0.72
HOMA-IR	2.2 (0.7)	2.3 (0.8)	0.77
Waist circumference (cm)	75.8 (5.6)	76.4 (6.5)	0.77
Hip circumference (cm)	98.5 (7.4)	98.7 (5.3)	0.93
Systolic blood pressure (mmHg)	120.8 (9.6)	120.8 (11.6)	0.99
Diastolic blood pressure (mmHg)	63.4 (8.2)	63.7 (6.8)	0.89
Total cholesterol (mmol/L)	3.9 (0.7)	4.1 (1.1)	0.55
LDL cholesterol (mmol/L)	2.2 (0.6)	2.3 (1.0)	0.55
HDL cholesterol (mmol/L)	1.4 (0.4)	1.3 (0.4)	0.53
Triglycerides (mmol/L)^‡^	0.8 (0.3–2.5)	0.9 (0.5–1.9)	0.24
HbA_1c_ (%) [mmol/mol]	5.2 (0.2) [33 (2.5)]	5.1 (0.3) [32 (2.8)]	0.15
Gestational age at birth (days)	259 (217–278) (*n*=18)	278 (258–301) (*n*=17)	< 0.001*
Birth weight (g)	3482 (711) (*n*=19)	3,571 (542) (*n*=18)	0.68
Maternal Characteristics			
Maternal age at birth (years)	28.6 (3.8) (*n*=20)	29.8 (3.7) (*n*=20)	0.30
Maternal pre-pregnancy BMI (kg/m^2^)	22.9 (2.3) (*n*=16)	24.2 (4.4) (*n*=17)	0.47*
Parity^#^	1.5 (1–3) (*n*=20)	1.8 (1–4) (*n*=18)	0.55*
Preeclampsia^€^	5 (26%) (*n*=19)	2 (12%) (*n*=17)	0.41^†^
Caesarean section (planned or emergency)	13 (68%) (*n*=19)	5 (28%) (*n*=18)	0.01*
Polyhydramnios	2 (11%) (*n*=19)	0 (*n*=17)	0.27^†^
Maternal HbA_1c_ (%) [mmol/mol]			
Pregestational	7.4 (1.3) [57] (*n*=18)	(–)	
1st trimester	7.2 (1.2) [55] (*n*=17)	(–)	
2nd trimester	6.3 (1.0) [45] (*n*=19)	(–)	
3rd trimester	6.5 (0.9) [48] (*n*=18)	(–)	
Maternal use of insulin (IE pr. day)			
Pregestational	39 (11) (*n*=19)	(–)	
1st trimester	40 (11) (*n*=17)	(–)	
2nd trimester	47 (14) (*n*=18)	(–)	
3rd trimester	59 (16) (*n*=18)	(–)	
Maternal hypertension^ψ^			
Pregestational	1 (7%) (*n*=16)	(–)	
1st trimester	1 (5%) (*n*=19)	(–)	
2nd trimester	2 (11%) (*n*=19)	(–)	
3rd trimester	7 (37%) (*n*=19)	(–)	
Maternal albuminuria^ϖ^			
Pregestational	4 (25%) (*n*=16)	(–)	
1st trimester	4 (21%) (*n*=19)	(–)	
2nd trimester	4 (24%) (*n*=17)	(–)	
3rd trimester	8 (42%) (*n*=19)	(–)	

Data are presented as mean and standard deviation, median, and range or as number and percent. *HOMA-IR* Homeostasis model for insulin resistance (Matthews et. al Diabetologia 28 412-419). ^€^Preeclampsia was defined as blood pressure>140/90 and proteinuria 2+ on a urine protein test strip (equal to 1.0 g/l). ^ψ^Hypertension in pregnancy was defined as blood pressure >140/90 or use of antihypertension medication prior to pregnancy. ^ϖ^Maternal albuminuria is defined as either microalbuminuria (30–300 mg/24 h) or macroalbuminuria (>300 mg/24 h). *P*-values are generated using Student’s *T* test, *Wilcoxon rank-sum test, or ^†^Fishers exact test. ^‡^Log-transformed, ^#^even though not being normally distributed, we display data as mean and range

### Clinical examination

All measurements except from height were performed three times, and the mean value was used for the analyses. We measured height to the nearest 0.1 cm and weight (in kilograms) to the nearest 0.1 kg. Waist circumference was measured using a tape measuring to the nearest 0.5 cm midway between arcus costae and crista iliaca after exhalation and hip circumference corresponds to the widest measure around the hips. Preeclampsia was defined as blood pressure > 140/90 mmHg and proteinuria 2+ on a urine protein test strip (equal to 1.0 g/l).

### Oral glucose tolerance test (OGTT)

We performed a standard 2-h OGTT by using a glucose load of 1.75 g/kg body weight up to a total of 75 g. Plasma glucose was measured at 0 and 120 min and serum insulin at 0 min (fasting).

Insulin sensitivity was evaluated by HOMA-IR and calculated using the original equation [21].

### Biochemical analyses

Glucose was measured in venous plasma with a hexokinase-glucose-6-phosphate dehydrogenase assay (Abbott Diagnostics, Abbott Park, Illinois, USA). Serum insulin was measured by ELISA using dual-monoclonal antibodies (ALPCO Diagnostics, Salem, New Hampshire, USA). Lipids were measured by enzymatic calorimetric analysis, end-up reaction (Abbott). Analyses of maternal HbA_1c_ between 1993 and 1999 were measured with local assays. Correction was made to a common standard (normal range of standard assay, 0.044–0.064) by multiplying the HbA_1c_ value with a correction factor as previously described (mean of the reference values for a standard assay divided by the mean of the reference values for the given assay) [19].

### Statistics

Continuous variables with symmetric distribution are presented as means and standard deviation (SD), continuous variable with skewed distribution as medians, and interquartile range (IQR) or range. Comparison of groups was performed using Student’s *T* test, Wilcoxon rank-sum test, chi-square, or Fisher’s exact test. Statistical analyses were done in STATA 13.1.

### DNA isolation and the 450K-Illumina Infinium assay

EDTA-treated peripheral blood samples were collected from the participants when they participated in the EPICOM study and were stored as EDTA treated at −80 °C until use. Genomic DNA was extracted from peripheral blood using QIAmp Mini Kit (Qiagen, Germany). For each sample, 1 μg of genomic DNA was bisulfite-converted using Zymo EZ DNA Methylation Kit according to the manufacturer’s recommendations. DNA methylation level was measured using the 450K-Illumina Infinium assay (Illumina, Inc.) at Aros Applied Biotechnology A/S.

### (Pre-)processing of the 450K-Illumina Infinium assay data

All analyses were performed in R statistics, version 3.6.1, and R package minfi (version 1.32.0) was used for normalization, analysis, and visualization [22]. Detection *p*-values were calculated to identify failed positions with a *p*-value cut-off > 0.01. Probes were removed if they failed in more than 20% of the samples (*n* = 350). No samples were identified as failed, as the proportion of failed probes did not exceed 1% for any single sample. Density bean plots were used to identify outliers, and minfi’s inbuilt function was used to evaluate data with respect to extreme methylation outliers (> 3 SD away from the median). We performed background normalization and control normalization implementing the pre-processing choices of Genome Studio. Next, we applied subset-quantile-within-array-normalization correcting for technical differences between Infinium type I and II assay design allowing both within-array and between-sample normalization [23]. Cross-reactive probes (*n* = 29.541), probes with SNPs documented in C or G of the target (*n* = 18.284), and probes on sex chromosomes (*n* = 12,312) were excluded, leaving 415,009 probes. Methylation values were calculated as *M*-values (logit [beta]) (Equation (I)) [24].

$$M- value=\mathit{\log}2\left(\frac{Beta}{1- Beta}\right)$$

Multidimensional scaling plots were evaluated to identify clusters of samples behaving differently than expected. Finally, the probes were annotated to the human genome version 19 using the enhanced Illumina annotation method developed by Price et al. [25].

### Estimated differential cell counts

We adjusted for differences in cell proportions using minfi’s estimateCellCounts that implements Houseman et al.’s regression calibration algorithm [26]. This algorithm returns relative proportions of CD4+ and CD8+ T-cells, natural killer cells, monocytes, granulocytes, and B-cells in each sample.

### Identifying differentially methylated positions (DMPs)

To identify positions where methylation is associated with intrauterine exposure to maternal T1DM, we fitted a linear model, which utilizes a generalized least squares model (lmFit of R package *limma*) allowing for missing values [27]. Significance was evaluated using F-test. The sample variances were estimated using an empirical Bayes approach with shrinkage towards the means. A Benjamini-Hochberg FDR below 0.05 was considered significant. We applied the model both without and with adjustment for estimated relative cell proportions (CD4+ and CD8+ T-cells, natural killer cells, monocytes, granulocytes, and B-cells).

### Identifying differentially methylated regions (DMRs)

We used DMRcate to identify any autosomal DMRs [28]. DMRcate identifies and ranks the most differentially methylated regions across the genome based on kernel smoothing of the differential methylation signal. The model performs well on small sample sizes and builds on the well-established *Limma* package, allowing us to incorporate estimated cell proportions as covariates. A FDR below 0.05 with a |Δ*M*-value| < 1 was considered significant.

### RNA sequencing (RNA-Seq) sample preparation

Blood samples were drawn using PAXgene Blood RNA Tubes and placed 2 h at room temperature, sequentially stored overnight at −20° before being stored at −80° until analysis.

### RNA-Seq library construction and sequencing

Whole transcriptome, strand-specific RNA-Seq libraries were prepared from total RNA using the Ribo-Zero Globin technology (Illumina, Inc.) for depletion of rRNA and globin mRNA followed by library preparation using the ScriptSeq technology (Illumina, Inc.). Depletion and library preparation were automated on a Sciclone NGS (Caliper, Perkin Elmer) liquid handling robot. The total RNA (1.7 μg per sample) was subjected to Baseline-ZERO DNase prior to depletion. Total RNA was purified using Agencourt RNAClean XP Beads before and after DNase treatment followed by on-chip electrophoresis on a LabChip GX (Caliper, Perkin Elmer) and by UV measurements on a NanoQuant (Tecan). Cytoplasmic and mitochondrial rRNA as well as globin mRNA were removed from 400 ng DNAse-treated total RNA using the Ribo-Zero Globin Gold Kit (Human/Mouse/Rat, Illumina, Inc.) following the manufacturer’s instructions, and quality of the depleted RNA was estimated on a LabChip GX (Caliper, Perkin Elmer). Synthesis of strand-specific RNA-Seq libraries were conducted using the ScriptSeq v2 kit (Illumina, Inc.) following the recommended procedure, and the qualities of the RNA-Seq libraries were estimated by on-chip electrophoresis (HS Chip, LabChip GX, Caliper, Perkin Elmer) of a 1 μL sample. The DNA concentrations of the libraries were estimated using the KAPA Library Quantification Kit (Kapa Biosystems). The RNA-Seq libraries were multiplexed paired-end sequenced on a NextSeq 500 (75 + 6 + 75 bp) using a high-output flowcell.

### RNA-Seq analysis

Paired de-multiplexed fastq files were generated using bclfastq (v.2.20 Illumina) and initial quality control was performed using FastQC. Adapter trimming was conducted using the GATK ReadAdaptorTrimmer tool followed by mapping to the human genome (hg19) in addition to transcripts from databases on non-coding RNAs (mirbase, mitranscriptome, rfam, snornabase, tjumirna, and trnascanse) using Bowtie and then further analyzed using Tophat, and Cufflinks and HTSeq-count (union method) [29, 30]. HTSeq-count (union method) was applied to produce raw counts which were then submitted for differential expression analysis in R using edgeR [31]. All non-informative features were filtered out by removing features with less than one count per million (CPM) in 39 samples removing 98,334 features, leaving 13,842 for downstream analysis. A generalized linear model was fitted, and *p*-values and log fold changes (Log2FC) were retrieved from the individual comparisons of index vs. controls. A Benjamini-Hochberg FDR below 0.05 was considered significant.

### Weighted gene correlation network analysis (WGCNA)

Weighted gene correlation network analysis (WGCNA, v1.70.3) was applied to identify co-expressed genes from the RNA-Seq data, and how these co-expressed gene modules associated with the cohort group-parameter or clinical traits. Outlier samples were detected using hierarchical sample clustering, removing one female index sample. A signed co-expression network was constructed using a one-step approach, calculating adjacency choosing an appropriate soft thresholding power with approximate scale-free topology. Gene clustering was performed on the signed Topology Overlap Matrix by hierarchical clustering, identifying modules via the blockwiseModules function with a minModuleSize of 30 and a mergeCutHeight of 0.25. The module eigengenes were calculated via the moduleEigengenes function, and eigengene significance and corresponding *p*-value were obtained for each module-trait association. Intramodular connectivity and gene significance was extracted for each module of interest and hub genes were identified using the chooseTopHubInEachModule function.

### Functional enrichment analysis

To investigate possible biological functions of the RNA expression changes in our cohort, we performed gene set enrichment analysis (GSEA) using Clusterprofiler [32]. The input genes were ranked based on the magnitude of changes in the index vs. controls comparison (log2FC). Disease associations were identified by disease ontology (DO) enrichment analysis, while Gene Ontology Biological Processes (GOBP) and REACTOME were used for functional and pathway enrichment. The top-enriched terms for each enrichment analysis were sorted according to *p*-value and presented as barplots. Furthermore, gene-concept networks that depict linkages of the genes and biological terms (DO, GOBP, and REACTOME terms) were made by evoking the cnetplot function of Clusterprofiler.

For methylation data, GOBP and REACTOME enrichment analysis were performed on all differentially methylated positions (*p* < 0.005) using the methylGSA package for R [33]. The probes were annotated to the human genome version 19 as previously described.

### Correlation between DNA methylation and gene expression

DNA methylation has been shown to play an important role in modulating gene expression. Therefore, we correlated changes in methylation and gene expression of the gene closest to the methylation site. Shared differentially expressed genes (DEGs) and DMPs (closest gene to methylation site) between index and controls (*p* < 0.05) were plotted in a scatter plot based on log2FC and Δ*M*-value. In the second part of the analysis, we used Spearman’s rank correlation to analyze the correlation between the specific methylation level (*M*-value) and RNA expression level (normalized counts) for shared genes. We considered the correlations to be interesting (either negative or positive) if *p* < 0.1.

### Analysis software

Statistics was done using R 3.6.1 (R Foundation for Statistical Computing, Vienna, Austria) with Bioconductor 3.9 [34]. DNA methylation data was analyzsed using the *minfi* [22], *DMRcate* [28], and *Limma* [35] package 1.32.0, and RNA-Seq data using edgeR [31]. Functional gene enrichment analysis was carried out using the R packages Clusterprofiler and MethylGSA [32, 33]. Graphics were made using basic R functions ggbio, Gviz, DEseq, DEXSeq, and ggplot2. The package knitr was used for data documentation.

## Results

Through medical records and the original Diabetes Association registry, we retrieved information regarding pregnancy and birth for the majority of both index and controls (Table 1). The index and controls included in the exploratory cohort were similar on most parameters including BMI, OGTT fasting plasma glucose, OGTT 120 min plasma glucose, fasting insulin, HOMA-IR, HbA_1c_, systolic and diastolic blood pressure, and birth weight, although significant differences were present in some of these parameters in the larger EPICOM group [20]. Gestational age at birth was significantly different for the intrauterine T1DM exposed children compared to the controls (at that time it was common practice to induce labor ~2–3 weeks prior to the expected delivery date in women with diabetes) (gestational age 259 (range=217–278) days vs. 278 (258–301) days, *p* <0.001), as was delivery by caesarean section (T1DM offspring 68% vs. 28% in the control group, *p*=0.01). Also, maternal age at birth, maternal pre-pregnancy BMI, risk of polyhydramnios, and parity were similar between the women with T1DM and the mothers of the controls. For most of the women with T1DM, we also had knowledge of their glycemic regulation pregestationally and during the pregnancy as well as average daily amount of insulin, hypertension status, and level of albuminuria. For the RNA-Seq data, one sample from the control cohort was missing. This however did not change the overall phenotypic results.

### Differentially methylated positions and regions

**Fig. 1:**
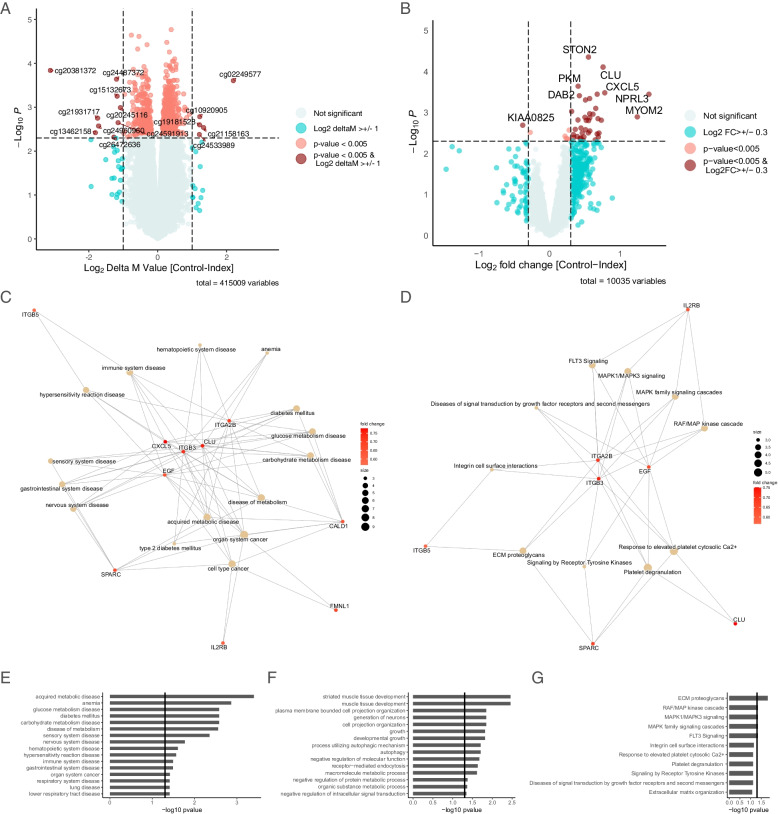
Differences in DNA methylation and gene expression between type 1 diabetes-exposed offspring and matched controls. **a** Volcano plot of −log10 *p*-value against delta-*M*-values of differentially methylated positions. Red dots denote DMPs between type 1 diabetes-exposed offspring and matched controls with a *p*-value < 0.005 and an absolute |Δ*M*-value| >1. The horizontal line represents *p*-value = 0.005. **b** Volcano plot of −log10 *p*-value against log fold change of differentially expressed coding genes. Red dots denote differentially expressed coding genes with a *p* < 0.005 and a log2FC ≥ 0.3. The horizontal line represents *p* = 0.005. **c–e** Functional enrichment analysis of the gene expression changes observed in type 1 diabetes-exposed offspring compared to matched controls. The differentially expressed coding genes were used as input for gene set enrichment analysis, to identify enriched disease phenotypes (DO) (**c**), biological processes (GOBP) (**d**), and biological pathways (REACTOME) (**e**). The top-enriched terms were sorted according to *p*-value (vertical black line denotes *p* = 0.05). A network plot (**f, g**) depicted the genes that were involved in the significant DO terms (**f**) and REACTOME terms (**g**)

Performing whole-blood genome-wide DNA methylation analysis using the 450K-Illumina Infinium assay, we identified 13,867 DMPs with a *p* < 0.05 between index and controls. To reduce the number of false positives, a criterion of *p* < 0.005 and |Δ*M*-value| > 1 was applied, resulting in 14 DMPs (9 hypomethylated and 5 hypermethylated) (Fig. 1a and Additional file 1: Table. T1) However, after correction for multiple testing, no single DMP reached the threshold of a Benjamini-Hochberg false detection rate (FDR) (< 0.05). DMRs defined as methylation in groups of nearby positions have been proposed to be involved in transcriptional regulation. Therefore, we extended our analysis to the regional level. Applying a *p* < 0.005, 37 DMRs were found (Additional file 2: Table T2). However, no DMRs were identified applying a FDR < 0.05.

### RNA expression

Subsequently, we analyzed gene expression by RNA-Seq to test if any DEGs, coding or non-coding RNA, were present when comparing our two cohort groups (index vs. controls). We identified 39 coding DEGs (38 upregulated and 1 downregulated) (Table 2, Fig. 1b and Additional file 3: Fig. F1) (*p* < 0.005 and a log2FC ≥ 0.3). In addition, we also found 20 non-coding DEGs (7 upregulated tags and 13 downregulated tags) (*p* < 0.005 and a log2FC ≥ 0.3) (Additional file 4: Table T3 online). No DEGs reached an FDR < 0.05.

**Table 2:** List of differentially expressed autosomal coding genes between type 1 diabetes-exposed offspring and controls

Gene ID	External gene name	Log2 fold change	Log CPM	***P***-value	Chromosome name	Start position	End position
ENSG00000103148.11	*NPRL3*	1.402	4.682	0.0003637	16	134273	188859
ENSG00000036448.5	*MYOM2*	1.238	3.216	0.001291	8	1993155	2113475
ENSG00000163735.6	*CXCL5*	0.778	3.821	0.0003347	4	74861359	74864496
ENSG00000120885.15	*CLU*	0.754	3.931	7.9319E−05	8	27454434	27472548
ENSG00000259207.3	*ITGB3*	0.729	5.136	0.001563	17	45331212	45421658
ENSG00000005961.13	*ITGA2B*	0.696	3.951	0.003207	17	42449548	42466873
ENSG00000184922.9	*FMNL1*	0.687	5.329	0.004019	17	43298811	43324687
ENSG00000161911.7	*TREML1*	0.682	1.937	0.001432	6	41117080	41122075
ENSG00000113140.6	*SPARC*	0.670	4.702	0.0003625	5	151040657	151066726
ENSG00000175746.4	*C15orf54*	0.670	2.299	0.003372	15	39542885	39547046
ENSG00000138798.7	*EGF*	0.650	3.438	0.0008035	4	110834040	110933422
ENSG00000122786.15	*CALD1*	0.642	2.518	0.004715	7	134429003	134655479
ENSG00000100385.9	*IL2RB*	0.610	3.173	0.001113	22	37521878	37571094
ENSG00000082781.7	*ITGB5*	0.566	3.489	0.002852	3	124480795	124620265
ENSG00000198478.6	*SH3BGRL2*	0.565	6.232	0.0013672	6	80341000	80413372
ENSG00000168497.4	*SDPR*	0.564	6.971	0.001083	2	192699028	192711981
ENSG00000151693.5	*ASAP2*	0.564	2.812	0.0004620	2	9346894	9545812
ENSG00000185909.10	*KLHDC8B*	0.549	2.620	0.001593	3	49209044	49213917
ENSG00000140022.5	*STON2*	0.547	4.040	4.5483E−05	14	81727000	81902809
ENSG00000019582.10	*CD74*	0.545	6.485	0.003180	5	149781200	149792492
ENSG00000101162.3	*TUBB1*	0.516	7.425	0.001578	20	57594309	57601709
ENSG00000090975.8	*PITPNM2*	0.506	2.549	0.002654	12	123468027	123634562
ENSG00000095303.10	*PTGS1*	0.505	5.532	0.0005005	9	125132824	125157982
ENSG00000111644.3	*ACRBP*	0.497	2.010	0.001323	12	6747241	6756626
ENSG00000169313.9	*P2RY12*	0.483	3.076	0.003989	3	151055168	151102600
ENSG00000163737.3	*PF4*	0.471	4.875	0.004818	4	74846794	74847841
ENSG00000141522.7	*ARHGDIA*	0.459	3.114	0.003189	17	79825597	79829282
ENSG00000054793.9	*ATP9A*	0.441	3.264	0.002419	20	50213053	50385173
ENSG00000107438.4	*PDLIM1*	0.440	3.137	0.004217	10	96997329	97050781
ENSG00000143368.9	*SF3B4*	0.429	2.080	0.004332	1	149895209	149900236
ENSG00000153071.10	*DAB2*	0.419	4.220	0.0003941	5	39371780	39462402
ENSG00000067225.13	*PKM*	0.401	6.571	0.0002313	15	72491370	72524164
ENSG00000143537.9	*ADAM15*	0.399	2.580	0.001539	1	155023042	155035252
ENSG00000146192.10	*FGD2*	0.387	5.061	0.003886	6	36973422	36996846
ENSG00000177830.13	*CHID1*	0.361	2.254	0.002612	11	867357	915058
ENSG00000157823.12	*AP3S2*	0.339	3.560	0.004107	15	90373831	90437574
ENSG00000075624.9	*ACTB*	0.335	9.389	0.003498	7	5566782	5603415
ENSG00000172757.8	*CFL1*	0.309	6.236	0.000952	11	65590493	65629497
ENSG00000185261.9	*KIAA0825*	−0.383	5.944	0.002067	5	93488671	93954309

Displayed are genes with a crude *p*-value<0.005 and a log fold change≥0.3

### Functional enrichment analysis

To gain mechanistic insight into functional importance of the coding DEGs found above, we used enrichment analysis to associate these changes to disease ontology (DO), biological processes (GOBP), and biological pathways (REACTOME). We observed enrichment in disease ontologies relating to acquired metabolic disease, glucose metabolism disease, diabetes mellitus, carbohydrate metabolism disease, disease of metabolism, and clustering around genes including *CXCL5*, *EGF*, and *SPARC* (Fig. 1c–g)*.* For biological processes and pathways, the analysis revealed enrichment relating to striated muscle tissue development, muscle tissue development, growth, and developmental growth and biological pathways including ECM proteoglycans, RAF/MAP kinase cascade, MAPK1/MAPK3 signaling, and MAPK family signaling (Fig. 1c–g and Additional file 5: Fig. F2 online).

Functional enrichment analysis was also carried out with identified DMPs (*p* < 0.005) as input. Again, enrichment in disease ontologies and biological pathways relating to carbohydrate metabolism, glucose transportation, and pathways including glucagon-like peptide-1 (GLP1) regulated insulin secretion were in the top-enriched terms (Additional file 6: Table T4 and Additional file 7: Table T5 online).

### Weighted gene correlation network analysis

**Fig. 2:**
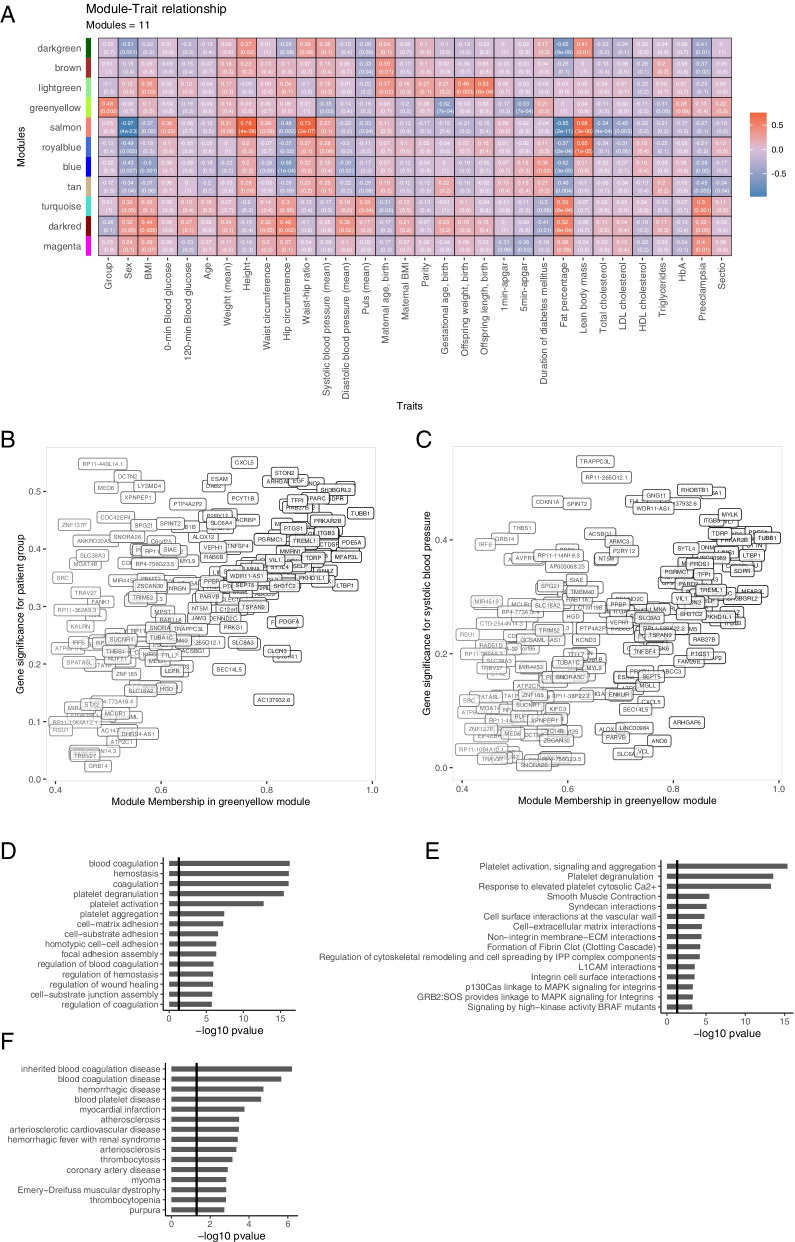
Weighted gene correlation network analysis (WGCNA) of all expressed genes. The genes were sorted into modules, with each module appointed a color, based on co-expression patters. **a** The eigengene of each module was correlated to the measured clinical traits. The correlation values and *p*-values were depicted for each correlation, which were colored based on positive (red) or negative (blue) correlation. **b** The module-membership vs gene significance for group (index vs control) for all genes within the greenyellow module. **c** The module-membership vs gene significance systolic blood pressure for all genes within the greenyellow module. **d–f** Functional enrichment analysis of the genes within the greenyellow module, to identify enriched biological processes (GOBP) (**d**) and biological pathways (REACTOME) (**e**) and disease phenotypes (DO) (**f**)

As we were unable to robustly identify changes at the single-gene level, we used WGCNA to detect modules of correlated genes, and to test if any of these modules could be related to our two cohort groups and the associated clinical traits. Based on co-expression similarities, the genes were split into 25 modules (Additional file 8: Fig. F3). The eigengenes of these modules, representing a summary of the expression of all genes within the module, were correlated with group status, index or control, and the clinical and paraclinical measurements. The modules that were significantly associated to our two cohort groups, sex or clinical traits, were selected (Fig. 2a). The strongest association observed was a negative correlation of the salmon module with the gender female (sex, cor = −0.97, *p* = 4e−23). This module consisted of 153 genes, and as expected, almost exclusively came from the sex chromosomes (21 from the X chromosome and 13 from the Y chromosome). Other female-specific traits like increased hip circumference and fat percentage were also negatively correlated with eigengene expression in this module, while male traits like increased height, waist-hip ratio, and lean body mass were positively correlated. Interestingly, we also observed that the greenyellow module, consisting of 201 genes, was positively correlated with the T1DM offspring group (cor = 0.48, *p* = 0.002) and unaffected by sex. We used a signed network structure to create our modules, and thus, a general upregulation of all genes within this module was expected for the index group. This assumption was supported by the presence of 22 out of the 38 upregulated DEGs in the greenyellow module. The association of the individual genes to the index group revealed that a subset of the module genes was more strongly correlated with the index group (e.g., CLU, SDPR, BEND2, EGF, STON2, TFPI, SPARC, TUBB1) (Fig. 2b). Furthermore, the greenyellow module was negatively correlated with offspring systolic blood pressure at follow-up (cor = −0.35, *p* = 0.03) (Fig. 2c), gestational age at birth (cor = −0.52, *p* = 7e−04) and the 5-min Apgar score (−0.53, *p* = 7e−04). Maternal BMI was not correlated to any modules; however, maternal age was positively correlated to the lightgreen module, which was also positively correlated to increased offspring weight and length. The presence of preeclampsia was not significantly associated to the greenyellow group module (T1DM offspring). However, we observed that preeclampsia was positively correlated to the magenta, darkred, and turquoise modules and negatively correlated to the tan, blue, brown, and darkgreen module. Interestingly, almost the exact same correlations were observed for fat percentage. Thus, presence of preeclampsia may predispose to an increased fat percentage later in life.

All genes from the greenyellow module were used as input for functional enrichment analysis to associate these changes to disease ontology (DO), biological processes (GOBP), and biological pathways (REACTOME) (Fig. 2d–f). Enrichment in ontologies and pathways relate to platelet degranulation/activation, blood coagulation, smooth muscle cell contraction, and fibrin clot formation. Within disease ontologies, we observed enrichment in terms relating to coagulation disease, atherosclerosis, thrombosis, myocardial infarction, and hemorrhagic disease.

### Correlation between DNA methylation and gene expression

**Fig. 3:**
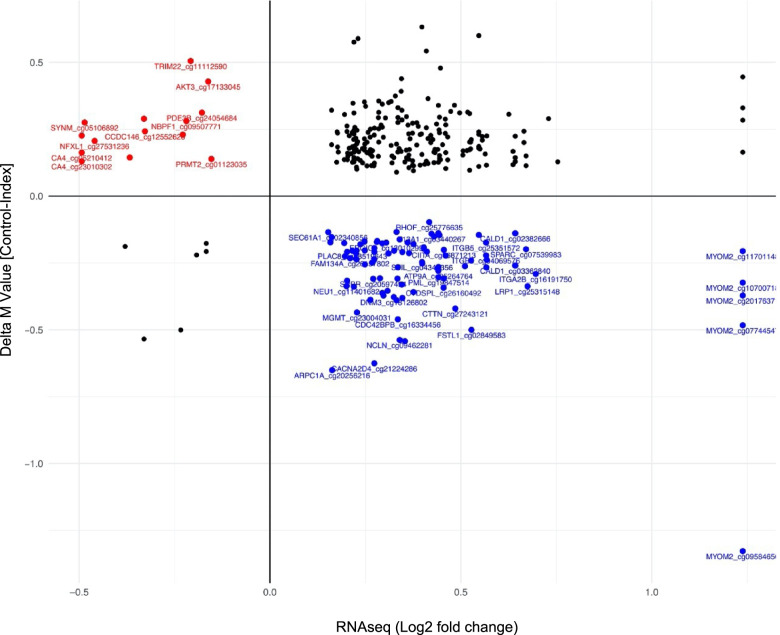
Correlated changes in DNA methylation and gene expression in type 1 diabetes-exposed offspring compared to matched controls. Scatter plot depicting mean methylation difference (delta-M) versus mean gene expression change (log2FC), of shared DEGs (*p* < 0.05) and DMPs (closest gene to methylation site, *p* < 0.05). Upper-left (red): Increased methylation corresponding to a decreased gene expression. Lower-right (blue): Decreased methylation corresponding to increased gene expression. Upper-right: Increased methylation corresponding to increased gene expression

DNA methylation has been shown to play a regulatory role in mediating gene expression, and if located within a gene promotor, higher levels of methylation usually repress transcriptional expression [8]. To further explore the consequences of altered DNA methylation patterns, we therefore compared the DNA methylation data with our gene expression data (Fig. 3 and Additional file 9: Table T6). First, we visualized correlations between changes at the group level. That is, the mean change in methylation level plotted against the mean change in gene expression of the gene annotated to the methylation site. DEGs overlapping with DMPs (annotated to closest gene to the methylation site), between T1DM exposed offspring and controls (*p*-value < 0.05), were plotted in a scatter plot based on log2FC and Δ*M*-value.

This analysis revealed that 93 DMPs annotated to 76 DEGs showed a pattern where DNA hypomethylation was associated with an increased RNA expression (Fig. 3, lower-right (blue)). Fourteen DMPs annotated to 12 DEGs were found to show a pattern were DNA hypermethylation was associated with decreased RNA expression (Fig. 3, upper-left (red)). Two hundred one DMPs annotated to 129 genes showed a pattern where DNA hypermethylation was associated with increased RNA expression. As the second step of our correlation analysis, we performed a Spearman rank correlation for each DMP-DEG pair, using each subject’s methylation level at the involved DMPs and expression level (normalized counts) for the corresponding RNA expression data as input. Here, our analysis showed that 2 DMPs and their corresponding genes had significant inverse correlation between DNA hypomethylation and increased RNA expression (*p* < 0.1) (*CIITA* and *TPM1*, Table 3). DNA hypermethylation of CpG sites localized within the gene body has been described as being associated with increased RNA expression, and in our analysis, we found 4 annotated DMPs localized within the gene body and their corresponding genes displaying positive correlation between DNA methylation and RNA expression (*p* < 0.1) (*PXN*, *ST8SIA1*, *LIPA*, and *DAXX*, Table 3).

**Table 3:** Top candidate genes from the two-step correlation analysis

	Probe name	Gene region	Relation to CPG island	Gene symbol	***rho***	***P***-value	FDR ***P***-value
DNA hypomethylation and positive RNA expression	cg06871213	Body	Nshore	*CIITA*	−0.3490	0.03002	0.06005
	cg04194852	TSS1500	Island	*TPM1*	−0.3237	0.04493	0.42438
DNA hypermethylation and positive RNA expression (located in gene bodies)	cg04256697	Body;TSS1500;Body	OpenSea	*PXN*	0.3933	0.01379	0.05514
	cg24112692	Body	OpenSea	*ST8SIA1*	0.3686	0.02152	0.26539
	cg13931663	Body;Body	OpenSea	*LIPA*	0.3020	0.06206	0.56834
	cg20618109	Body;Body;TSS1500;Body;Body	N_Shore	*DAXX*	0.2909	0.07271	0.581641

Correlation of DNA methylation and RNA expression between type 1 diabetes-exposed and controls. Rho is generated from Spearman’s rank correlation analyzing the correlation between the specific methylation level (*M*-value) and log fold change for shared genes. FDR-adjusted *P*-value generated using Benjamini-Hochberg correction

## Discussion

This exploratory study suggests the existence of epigenetic marks (e.g., DNA methylation) that possibly influence the long-term health of offspring born to women with pregestational T1DM and elucidate intuitive pathways to be involved. Combining DNA methylation with gene expression data in offspring exposed to T1DM in utero is novel and provides new insight into the consequences of subtle epigenetic changes on gene expression and disease. Although none of the DMPs and DEGs remained significant after correction for multiple testing, our WGCNA indicated that subtle alterations in gene expression networks were present and may have a biological significance.

We identified 14 DMPs (*p* < 0.005 and |Δ*M*-value| > 1), 9 being hypomethylated and 5 hypermethylated, between T1DM exposed offspring and their matched unexposed controls. We also sought to identify any overall differences in RNA expression between index and controls and were able to identify a set of 39 predominantly upregulated genes (*p* < 0.005 and a log2FC ≥ 0.3). This set of upregulated genes was further validated by WGCNA. Here, 25 modules of co-expressed genes were identified, and one of these modules was positively correlated with the index group. A significant part of the genes in this module overlapped with the identified DEGs. Interestingly, this module was also negatively correlated to offspring systolic blood pressure at follow-up, gestational age at birth, and Apgar score at 5 min. As gestational age and Apgar score were generally lower for the index group, this seemed reasonable. Surprisingly, increased eigengene expression in this module, as observed in the index group, correlated with lower systolic blood pressure. From the enrichment analysis of the genes in this module, pathways and disease ontologies related to platelet activation and coagulation were among the top-enriched. Thus, vasodilation may be a consequence of the gene expression changes observed, and over time, this may lead to a malfunctioning vasculature resulting in some of the long-term pathologies observed in offspring born to women with T1DM [36].

The functional enrichment analysis of the DEGs showed clustering around the genes *CXCL5*, *EGF*, and *SPARC*, which are associated with diabetes, obesity, and insulin secretion and enriched in disease ontology terms of metabolic disease, diabetes, and glucose metabolism disease. These findings seem to fit the hypothesis of altered intrauterine programming of offspring born to women with T1DM [37–39]. The functional enrichment analysis points towards muscle tissue development and developmental growth to be associated with intrauterine exposure to T1DM and that the MAPK signaling pathway could play a role in the pathogenesis. The link between intrauterine exposure to T1DM and later in life alterations in skeletal muscle has previously been described by Houshmand-Oeregaard et al., and even though no studies have described a link between intrauterine exposure to diabetes and later in life alterations in the MAPK pathway, the central placement of MAPK in insulin signaling makes it a plausible object for pathogenetic changes [14, 40].

Our access to both DNA methylation data and gene expression data made it possible to explore the impact of epigenetic changes (e.g., DNA methylation) induced by intrauterine T1DM exposure on gene expression. We chose to perform a two-step analysis where we first explored the overall correlation between DMPs and DEGs at the group level. During step 2, we studied the individual correlation between Δ*M*-value at the involved DMP and log2FC for the corresponding DEG. No studies within the area of fetal programming as a consequence of maternal T1DM have to our knowledge performed a similar linkage study between DNA methylation and RNA expression data. However, in cancer studies, a similar methodology has been used [41]. Applying this method provided us with six genes where changes in DNA methylation between offspring exposed to maternal T1DM and controls correlated with relevant changes in gene expression (Table 3). *PXN*, which is localized at chromosome 12, has been linked to progression of T1DM and *LIPA* at chromosome 10 to the composition of lipoproteins [42, 43]. Mutations in *TPM1* at chromosome 15 is associated with both the development of the metabolic syndrome and cardiac hypertrophy [44, 45]. *CIITA* is associated with MHC class II (MHCII) expression and MHCII antigen presentation in adipocytes and is reported to trigger early adipose inflammation and insulin resistance as well as inducing changes to energy expenditure in skeletal muscle [46, 47].

In our study, we chose to match our participants so that they did not overtly differ in phenotypic appearance. This approach resulted in a very homogenous group where the major difference was in T1DM exposure status. The matching was an attempt to exclude any phenotypic differences to be the cause of differences in methylation status. However, a consequence of the matching could, in theory, result in the observed changes in methylation and gene expression as being protective towards the development of later in life metabolic disease, as none of the participants in the exploratory cohort had overt metabolic disease in contrast to the participants in the full EPICOM study [20]. Also, it is well known that gestational age is associated with DNA methylation, and as gestational age differed between our two cohorts, this could have influenced our findings [48].

Epigenetic changes in relation to intrauterine exposure to either nutrition or maternal diabetes has previously been described in animal studies but the results have been difficult to apply to human studies [49, 50]. In recent years, methylation analyses of cord blood have shown promising results, but the use of cord blood methylation status as a biomarker of long-term offspring risk of disease development has yet to be fully described [11, 51].

Here we used leucocytes from peripheral blood from both T1DM exposed and non-exposed offspring. The most likely target tissue for the pathogenesis of long-term consequences of being born to a mother with T1DM may, however, be pancreatic β-cells, and muscle and fat tissue. These three tissues are not as readily available, and it is debated whether peripheral blood leucocytes reflect global epigenetic changes induced by maternal pregestational T1DM. Studies of tissue-specific differentially methylated regions have shown conservation of DNA methylation patterns across different tissues [52, 53]. However, replication of our findings in target tissues would further strengthen our hypothesis. In our study, we did not have access to specific white cell counts for each participant. Instead, we used the EstimateCellCounts algorithm from the minfi package, which enables us to minimize confounding related to cell composition, a strength compared to prior studies within the same area.

The samples used in our study were collected at a mean age of ~18 years. This provides time to develop phenotypic characteristics. However, it also provides the possibility that any epigenetic changes have occurred during the time period from birth to follow-up. In the EPICOM study, we did not have access to cord blood samples and it is therefore not possible for us to state that the epigenetic findings in our study have been present since birth. Lifestyle and diet in families where the mother has T1DM could be different compared to families without diabetes and this could affect the epigenetic findings [54, 55].

When interpreting our finding, one must consider the fact that no single DMP reached the FDR-adjusted value of 0.05. This is probably a consequence of our modest sample size in the exploratory cohort and an obvious limitation of our study. Instead, we used the same pragmatic threshold for DMPs as West et al. providing an opportunity to compare findings in related studies [56]. Our limited sample size also hindered a robust exploration of maternal preeclampsia or being born either small or large for gestational age and later in life epigenetic and transcriptomic alterations. Interestingly, the WGCNA analysis did indicate a correlation between the presence of preeclampsia and an increased fat percentage later in life. This, however, was not associated to the T1DM group.

The strength of this study is that it was performed on a group of prospectively studied offspring born to women with T1DM. For both diabetes-exposed offspring and their matched controls, we have extensive knowledge of intrauterine exposure, birth, neonatal period, and current health status. Our women with T1DM are well characterized, and no women with type 2 diabetes (T2DM) or gestational diabetes (GDM) were included in our cohort. This enables us to explore associations between both fetal and long-term consequences of being born to mother with T1DM and epigenetic and transcriptomic alterations. However, access to a control group of offspring of mothers with GDM or T2DM would have benefitted our study and rendered possible a study of which epigenetic and transcriptomic alterations is induced by maternal hyperglycemia and which is induced by e.g., maternal pre-pregnancy overweight.

## Conclusions

Our data indicate intrauterine exposure to maternal T1DM to impact later in life methylation and gene expression in the offspring, a profile that may be linked to the increased risk of metabolic and vascular disease. However, comprehensive follow-up studies using a genome-wide approach and including relevant target tissue are needed.

## Supplementary Information

**Additional file 1: Table T1.** List of differentially methylated positions between type 1 diabetes exposed offspring and controls. Displayed are positions with a crude *p* < 0.005 and a M-value ≥ 1. The mean M values and mean B values displayed are corrected for cell count and age.

**Additional file 2: Table T2.** List of differentially methylated regions between type 1 diabetes exposed offspring and controls. Displayed are regions with a crude *p* < 0.005.

**Additional file 3: Figure F1.** Boxplots illustrating differences in CPM (Count Per Million) at each differentially expressed gene with a crude *p* < 0.005 between type 1 diabetes exposed offspring and controls.

**Additional file 4: Table T3.** List of differentially expressed non-coding RNA between type 1 diabetes exposed offspring and controls. Displayed are genes with a crude *p* < 0.005 and a log2FC ≥ 0.3.

**Additional file 5: Figure F2.** Network plots depicting the genes that are involved in the significant GOBP terms from the Functional Enrichment Analysis of the gene expression changes observed in type 1 diabetes exposed offspring compared to matched controls.

**Additional file 6: Table T4.** Gene Set Enrichment Analysis of differentially methylated positions. List of biological processes (Gen Ontology, GOBP) associated with the differentially methylated positions (*p* < 0.01).

**Additional file 7: Table T5.** Gene Set Enrichment Analysis of differentially methylated positions. List of biological pathways (REACTOME) associated with the differentially methylated positions (*p* < 0.01).

**Additional file 8: Figure F3.** Weighted gene correlation network analysis (WGCNA) of all expressed genes. The genes were sorted into modules, with each module appointed a color, based on co-expression patters. The eigengene of each module was correlated to the measured clinical traits. The correlation values and *p*-values were depicted for each correlation, which were colored based on positive (red) or negative (blue) correlation.

**Additional file 9: Table T6.** Correlation between DNA methylation and RNA expression between type 1 diabetes-exposed offspring and controls. Displayed are all correlations reaching a *p* < 0.05 and marked with grey are correlations reaching a *p* < 0.10 using Spearman Ranks correlation between level of methylation and gene expression (normalized counts). *P*-value (correlation analysis) and FDR (correlation analysis) is the *p*-value/FDR for the DEG used as input for the correlation analysis.

## Data Availability

The dataset supporting the conclusions of this article is available in the bmcEGA MOMA repository (https://ega-archive.org/dacs/EGAC00001000145) and will be available upon request within the General Data Protection Regulation.

## Abbreviations

BMI: Body mass index
DEGs: Differentially expressed genes
DMPs: Differentially methylated positions
DMRs: Differentially methylated regions
DO: Disease ontology
EPICOM: Epigenetic, Genetic and Environmental Effects on Growth, Metabolism and Cognitive Functions in Offspring of Women with Type 1 Diabetes
FDR: False detection rate
GDM: Gestational diabetes
GLP1: Glucagon-like peptide-1
GOBP: Gene Ontology Biological Processes
GSEA: Gene set enrichment analysis
IQR: Interquartile range
OGTT: Oral glucose tolerance test
SD: Standard deviation
T1DM: Type 1 diabetes
T2DM: Type 2 diabetes
WGCNA: Weighted gene correlation network analysis
