# Supplementary figures and images for: Epigenetic and transcriptomic alterations in offspring born to women with type 1 diabetes (the EPICOM study)
Source: BMC Med. 2022 Sep 23;20:338. doi: 10.1186/s12916-022-02514-x (PMC9503228; doi:10.1186/s12916-022-02514-x)

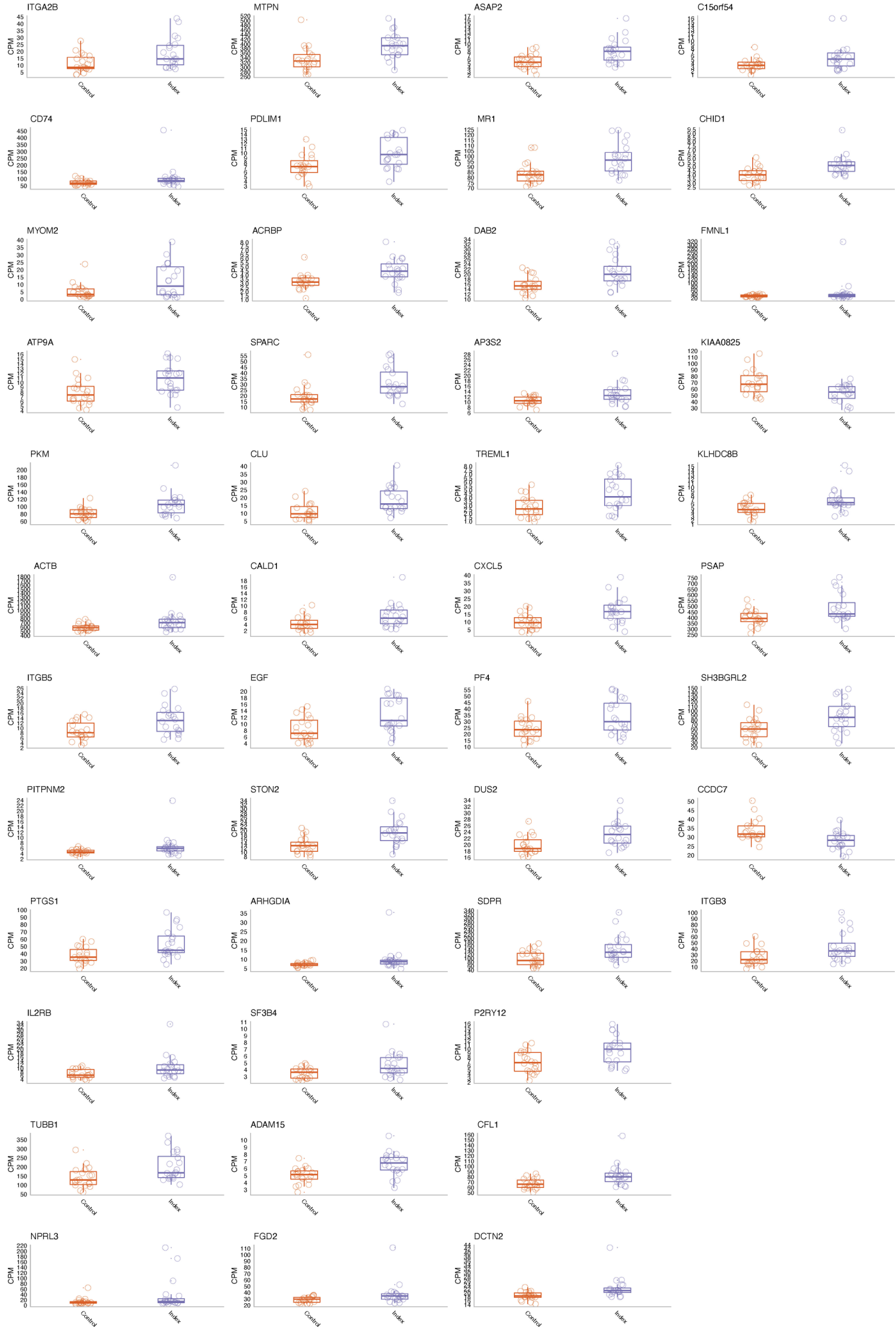

Supplement: Supplementary file 3 — Additional file 3: Figure F1. Boxplots illustrating differences in CPM (Count Per Million) at each differentially expressed gene with a crude p < 0.005 between type 1 diabetes exposed offspring and controls. [file 12916_2022_2514_MOESM3_ESM.pdf]

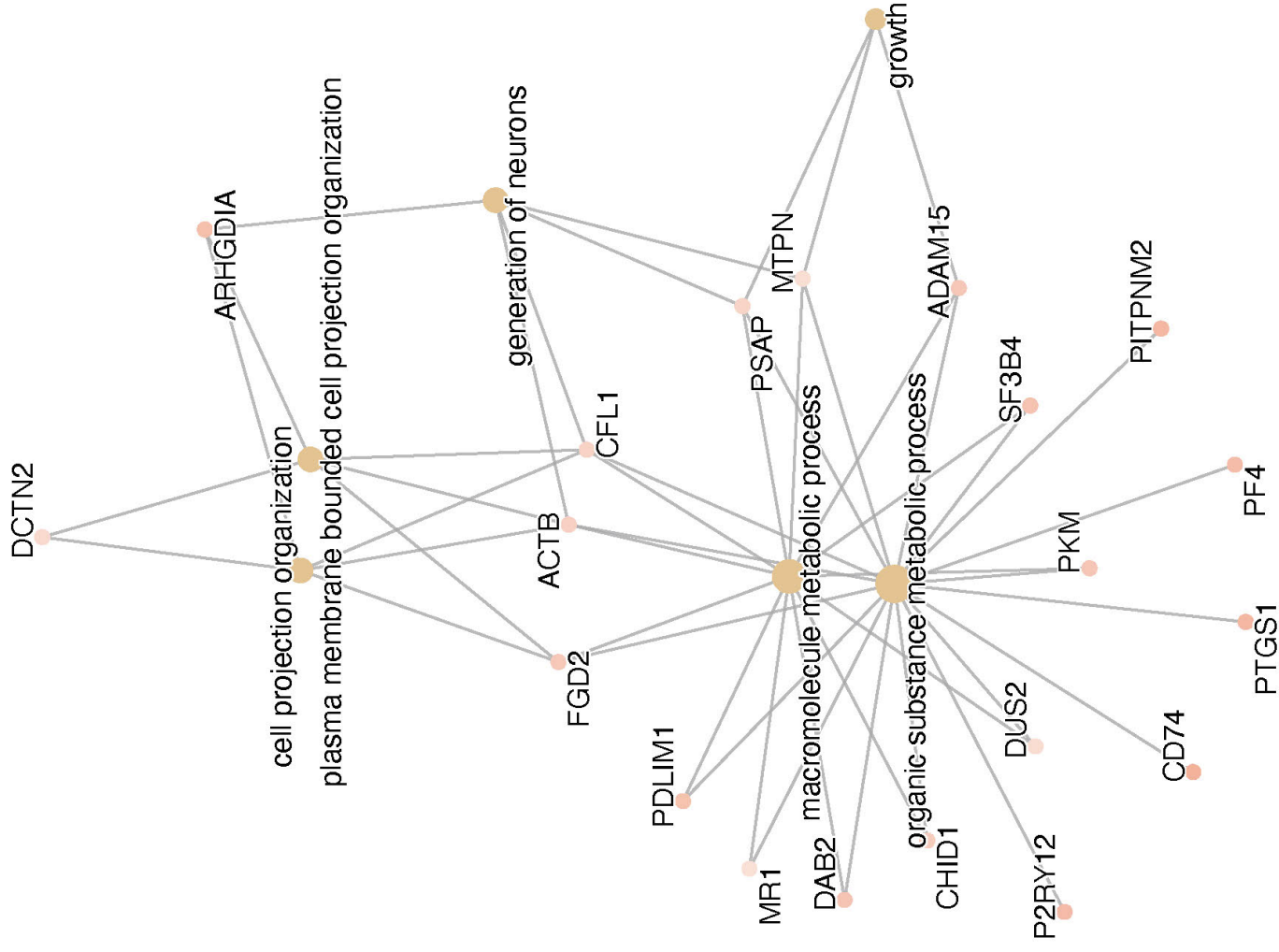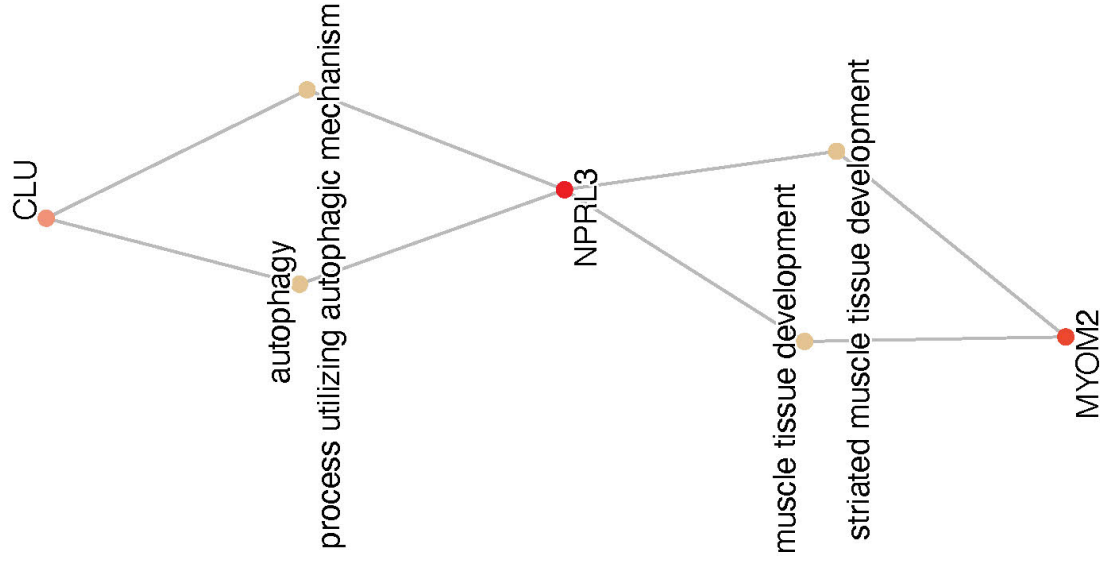

Supplement: Supplementary file 5 — Additional file 5: Figure F2. Network plots depicting the genes that are involved in the significant GOBP terms from the Functional Enrichment Analysis of the gene expression changes observed in type 1 diabetes exposed offspring compared to matched controls. [file 12916_2022_2514_MOESM5_ESM.pdf]

Module-Trait relationship

Modules = 25

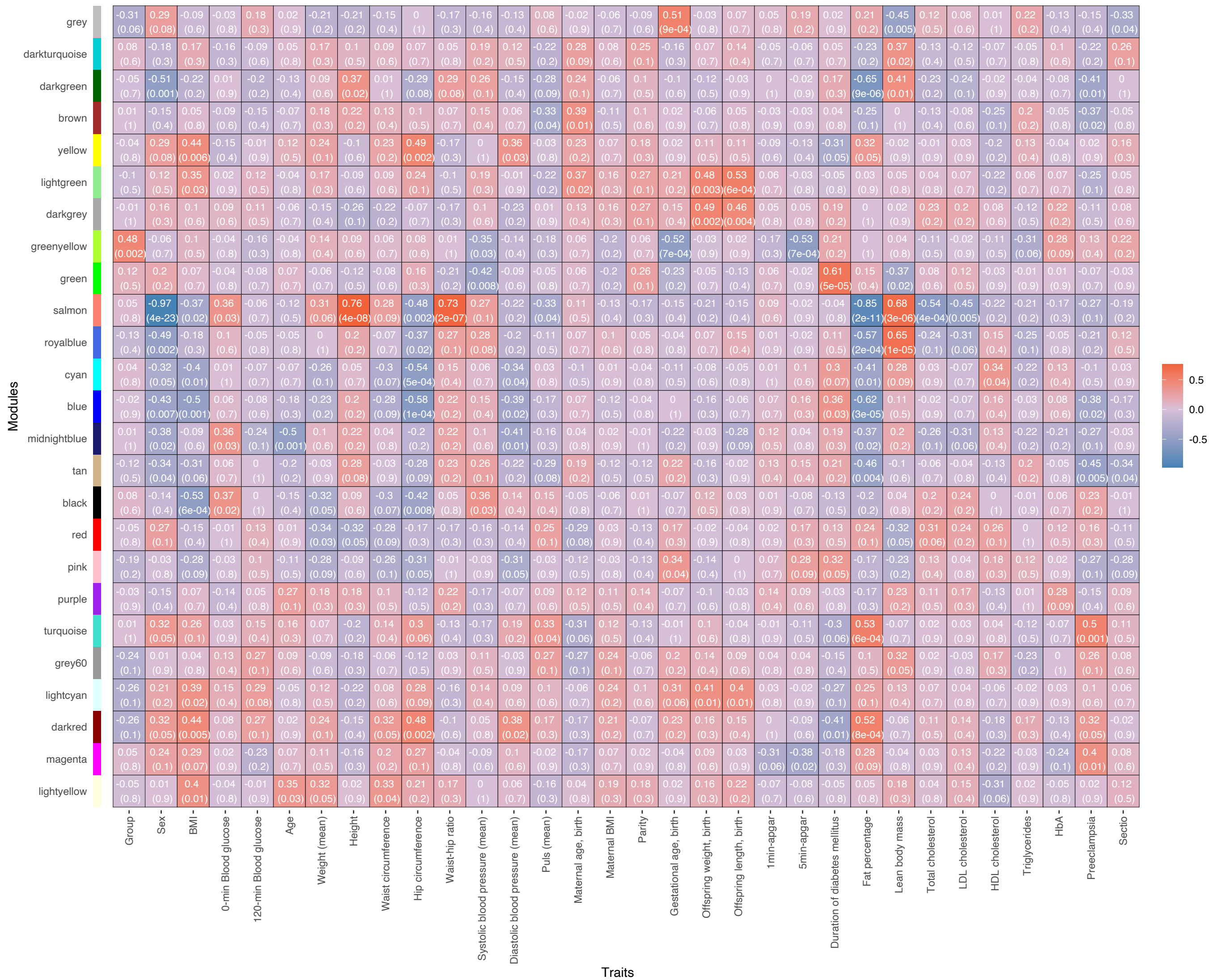

Supplement: Supplementary file 8 — Additional file 8: Figure F3. Weighted gene correlation network analysis (WGCNA) of all expressed genes. The genes were sorted into modules, with each module appointed a color, based on co-expression patters. The eigengene of each module was correlated to the measured clinical traits. The correlation values and p-values were depicted for each correlation, which were colored based on positive (red) or negative (blue) correlation. [file 12916_2022_2514_MOESM8_ESM.pdf]
